# The neural substrates of response inhibition to negative information across explicit and implicit tasks in GAD patients: electrophysiological evidence from an ERP study

**DOI:** 10.3389/fpsyg.2015.00275

**Published:** 2015-03-20

**Authors:** Fengqiong Yu, Chunyan Zhu, Lei Zhang, Xingui Chen, Dan Li, Long Zhang, Rong Ye, Yi Dong, Yuejia Luo, Xinlong Hu, Kai Wang

**Affiliations:** ^1^Laboratory of Cognitive Neuropsychology, Department of Medical Psychology, Anhui Medical UniversityHefei, China; ^2^Department of Neurology, The First Affiliated Hospital of Anhui Medical UniversityHefei, China; ^3^Anhui Mental Health CenterHefei, China; ^4^Institute of Social and affective Neuroscience, Shenzhen UniversityShenzhen, China

**Keywords:** general anxiety disorder, go/no-go task, response inhibition, N2, source localization

## Abstract

**Background:** It has been established that the inability to inhibit a response to negative stimuli is the genesis of anxiety. However, the neural substrates of response inhibition to sad faces across explicit and implicit tasks in general anxiety disorder (GAD) patients remain unclear.

**Methods:** Electrophysiological data were recorded when subjects performed two modified emotional go/no-go tasks in which neutral and sad faces were presented: one task was explicit (emotion categorization), and the other task was implicit (gender categorization).

**Results:** In the explicit task, electrophysiological evidence showed decreased amplitudes of no-go/go difference waves at the N2 interval in the GAD group compared to the control group. However, in the implicit task, the amplitudes of no-go/go difference waves at the N2 interval showed a reversed trend. Source localization analysis on no-go/N2 components revealed a decreased current source density (CSD) in the right dorsal lateral prefrontal cortex in GAD individuals relative to controls. In the implicit task, the left superior temporal gyrus and the left inferior parietal lobe showed enhanced activation in GAD individuals and may compensate for the dysfunction of the right dorsal lateral prefrontal cortex.

**Conclusion:** These findings indicated that the processing of response inhibition to socially sad faces in GAD individuals was interrupted in the explicit task. However, this processing was preserved in the implicit task. The neural substrates of response inhibition to sad faces were dissociated between implicit and explicit tasks.

## Introduction

General anxiety disorder (GAD) is the most persistent, severe, and prevalent class of mental disorder. This disorder is characterized by excessive worry, anxiety, and tension in addition to other somatic symptoms (Wittchen, 2002). Some studies have shown that anxiety is often associated with impaired cognitive control and avoidance behavior (Sehlmeyer et al., 2010). Response inhibition is an important component of this cognitive control system. Indeed, it has been established that an inability to inhibit a response to negative stimuli is linked to anxiety (Pacheco-Unguetti et al., 2012).

Response inhibition refers to inhibiting inappropriate behavior according to current task demands (Sehlmeyer et al., 2010). The typical paradigm of response inhibition is a go/no-go task. The different cognitive processing elements of response inhibition are conflict monitoring and action inhibition. Event-related brain potentials (ERPs), from recording electroencephalograms time-locked to stimuli, are a non-invasive brain imaging method. The main advantage of this method is high time resolution and thus can manifest the time course of brain activity. The methods have been frequently used to explore the neural substrates of response inhibition (Fabiani et al., 2000). It is believed that no-go-related N2 is related to conflict-monitoring processes, while no-go-related P3 is related to conflict resolution and behavior inhibition (Donkers and van Boxtel, 2004; Kropotov et al., 2011). Many neuroimaging studies have indicated that the right inferior prefrontal cortex (rIFC) plays a crucial role in the neural substrate of response inhibition to emotional stimuli (Ochsner et al., 2004; Shafritz et al., 2006; Goldstein et al., 2007; Berkman et al., 2009; Padmala and Pessoa, 2010). Previous studies have reported that anxious individuals exhibit a response inhibition deficit. An ERP study in children that used a modified emotional go/no-go task detected that when asked to suppress facial stimuli, anxious children showed decreased no-go/go differences in wave amplitudes during the N2 stage, and no-go/N2 amplitudes for calm faces predicted self-reported anxiety levels (Hum et al., 2013). Another study found that no-go/N2 was negatively related to anxiety-related personality traits (Sehlmeyer et al., 2010). These results suggested that dysfunctional response inhibition may be a trait indicator of anxiety. Although anxiety appears to disrupt inhibition, the neural correlates of this effect are far less understood. For example, anxiety may be due to a reduced ability to monitor conflict or may equally be linked to a reduced ability to apply active inhibition (Botvinick et al., 2001; Aarts and Pourtois, 2010; Berggren and Derakshan, 2013).

According to previous studies, behavioral and neural evidence confirmed that anxious individuals show an attention bias to negative faces, which results in greater negative emotional impulse strength and negative expressivity and reactivity to their emotions (Semlitsch et al., 1986; Decker et al., 2008; Roemer et al., 2009). The high arousal of negative emotions critically interrupts cognitive function, such as attention, memory, decision-making, executive function, and response inhibition ability (Salters-Pedneault et al., 2006; Mantella et al., 2007; Sass et al., 2010; Sehlmeyer et al., 2010; Paulus and Yu, 2012). Negative attention bias refers to the act of automatically fixing attention to negative aspects of internal or external events. ERP studies have reported shorter latency and larger amplitudes of P1, which were generated by extrastriate visual areas and index early attention processing in anxious compared to control individuals (Vuilleumier and Pourtois, 2007; Peschard et al., 2013). Another early ERP component that relates to top-down attention resources in face processing are N170 and the vertex positive potential (VPP; Ofan et al., 2013; Peschard et al., 2013). It has been reported that anxious individuals exhibit enhanced N170 and VPP activity when processing negative facial expressions (Frenkel and Bar-Haim, 2011; Ofan et al., 2013).

Sad facial expressions are one kind of fundamental negative emotional stimuli that convey important information in social communications (Schneider et al., 1994; Luo et al., 2010). Emotions induced by sad facial expressions influence an individual’s ability to inhibit inappropriate behavior. Many psychiatric individuals showed disabilities when regulating the relationship between sad facial information and response inhibition (Gehricke and Shapiro, 2000; Dziobek et al., 2011; Duerden et al., 2013; Hummer et al., 2013). When new mothers had more sad expressions, their infants expressed less joy and spent more time in joint negative affective states (Termine and Izard, 2009). Indeed, sad emotion is also associated with anxiety traits. When presented with sad facial stimuli, general social anxiety disorder patients showed hyperactivity in the medial frontal cortex extending into the anterior cingulated cortex (Labuschagne et al., 2012). However, few studies have focused on the relationship between sad emotion and anxiety.

In social situations, effortful explicit interpretation of the meaning of facial expressions may be required to guide an individual’s social responses. Yet, in familiar situations, facial expression encoding implicitly may also affect behavior without full cognitive awareness. Explicit processing means that facial expression is within the voluntary attention scope and is directly processed, whereas implicit processing means that facial expression is within the involuntary attention scope, and therefore is incidentally processed. Thus, the attention resource for stimuli processing is distinct between the two conditions. In fact, implicit and explicit facial processing serve different functions (Taylor et al., 2003) and have distinct neural substrates (Winston et al., 2003; Linden et al., 2010; Valdes-Conroy et al., 2014). It has been confirmed that facial expression processed explicitly and implicitly induced distinct emotional intensity in subject reports. Rating pictures was associated with significantly less intensity of sadness than passively viewing pictures, likely because the rating task reduced the activation of related brain regions responsible for an emotional experience (Taylor et al., 2003). Thus, faces processed implicitly and explicitly may showed dissociated effects on response inhibition. Our previous studies revealed that response inhibition was modulated by sad facial information at the action inhibition stage when facial expressions were processed explicitly rather than implicitly (Yu et al., 2014). However, until now, whether GAD patients show a deficit in response inhibition to sad faces across implicit and explicit conditions remains unclear.

The aim of the present study was to investigate the neural substrates of response inhibition to sad faces in GAD individuals across explicit and implicit tasks in a time course using ERP methods. We developed a modified emotional go/no-go task. In the task-relevant situation, subjects make their go/no-go decision according to the recognition of emotional categories; i.e., the emotional information was explicitly processed. In the task-irrelevant situation, subjects respond or inhibit their response based on the identification of the gender of the face, i.e., the emotional processing was implicit. Furthermore, in the explicit or implicit tasks, we used the same set of stimulus to preclude the distractions due to additional stimulus. A combination of ERP and source localization methods was used in order to further characterize the temporal and spatial characteristics of emotional response inhibition in implicit and explicit tasks in the GAD compared to normal groups. Based on previous studies indicating that GAD individuals exhibit attention bias to negative stimuli and dysfunction of response inhibition to negative stimuli, we hypothesized GAD individuals would show shorter latency and larger amplitudes in early ERP components, such as P1, N170, and VPP. More importantly, GAD individuals showed poor behavior performance and abnormal N2 and P3 components, as well as inhibition-related brain areas, such as the rIFC, in both implicit and explicit tasks.

## Materials and Methods

### Participants

Thirty-eight participants took part in the present experiment. Nineteen right-handed adults (nine female) aged 30.3 ± 7.3 (mean ± SD) years participated in the study from the Anxiety Disorders Clinic in Anhui Mental Health Center. Diagnostic assessments were completed by two psychiatrists according to DSM-IV. All GAD patients scored above 14 using the Hamilton Anxiety Rating Scale [HAMA-14]. The exclusion criteria included: (a) an IQ less than 85, according to the Wechsler Abbreviated Scale of Intelligence (WASI); (b) a current or lifetime history of psychotic disorder, bipolar disorder, pervasive developmental disorder, substance abuse, or eating disorder; (c) concurrent behavioral or drug treatments for anxiety; and (d) chronic medical illness that required a daily medication regime. Twenty age-matched adults screened for current and past psychiatric and neurological disorders were recruited through local advertisements. One woman in the control group was excluded from the analysis due to poor recording quality, leaving 19 for the final analysis (nine females). The matched group scored within the normal range on the HAMA-14. All participants had normal or corrected-to-normal vision. **Table 1** presents the background tests and characteristics of the final sample of clinical and comparison groups. All participants signed an informed consent form for the experiment. This study was approved by The Ethics Committee of Anhui Medical University.

**Table 1:** Group characteristics of the GAD group and CON group.

	GAD group	CON group	Between group comparison
	
	Mean (SD)	Mean (SD)	*P*-value
Sex (male/female)	9/10	9/10	0.87
Age (years)	30.3 (7.3)	26.6 (7.6)	0.13
Education (years)	12.9 (3.4)	13.5 (1.9)	0.49
Handedness (R/L)	19/0	20/1	0.32
HAMA	25.4 (7.1)	6.1 (1.2)	< 0.001

*GAD, general anxiety disorder; CON, control; HAMA, Hamilton Anxiety Rating Scale*.

### Stimuli

We selected 40 sad and 40 neutral faces from the native Chinese Facial Affective Picture System, including 20 female and 20 male faces displaying each emotion type. The faces in the Chinese Facial Affective Picture System were assessed with a 9-point scale by 100 college students from two colleges in Beijing and have been used in other studies (Luo et al., 2010). The stimulus for the present experiment were selected in such a way that they differed significantly in valence from one another (*t* = 11.65, *P* < 0.001 [*M* ± SD, sad: 3.11 ± 0.63, neutral: 4.49 ± 0.41]), but were similar in arousal (*P* > 0.5). The stimulus were similar to one another in size, background, spatial frequency, contrast grade, brightness, and other physical properties. Each picture was cropped into the shape of an ellipse that incorporated the facial characteristics using Adobe Photoshop 8.0^®^ software. The screen resolution was 72 pixels per inch, and the viewing angle was 5.7 × 4.6^°^. The subjects were seated in a soundproof room with their eyes approximately 100 cm from a 17-in screen. All stimuli were displayed in the center of the screen.

### Experimental Procedures

The experimental procedure was similar to that of previous studies (Yu et al., 2014). The present experiment included two types of emotional go/no-go paradigms: implicit task and explicit task. During the implicit task, the participants were instructed to respond after the presentation of faces depicting one gender (go trials) and to give no response after the other gender (no-go trial). In the explicit task, we asked the participants to respond or inhibit their behavior according to the valence of the facial expression. The implicit task and explicit task were presented in two separate parts, and the order of the two parts was counterbalanced across participants. Furthermore, each parts was sub-divided into two blocks in which the facial stimuli were counterbalanced in terms of whether they indicated go or no-go trials. Thus, the experimental procedure included four blocks that is negative-go/neutral-nogo and neutral-go/negative-nogo in explicit task, female-go/male-nogo, and male-go/female-nogo in implicit task.

Each block consisted of 480 trials that include 144 no-go stimuli and 336 go stimuli (30% vs. 70%). In each block, the go and no-go stimuli were presented pseudo-randomly, and a no-go trial was always preceded by a go trial. This was done in order to induce pre-potent motor responses and obvious conflict during response inhibition. At the start of each block, an instruction screen was presented for 5 min that prompted the participants to press or refrain from pressing the “J” key with their right hand according to the facial expression or gender.

**FIGURE 1:**
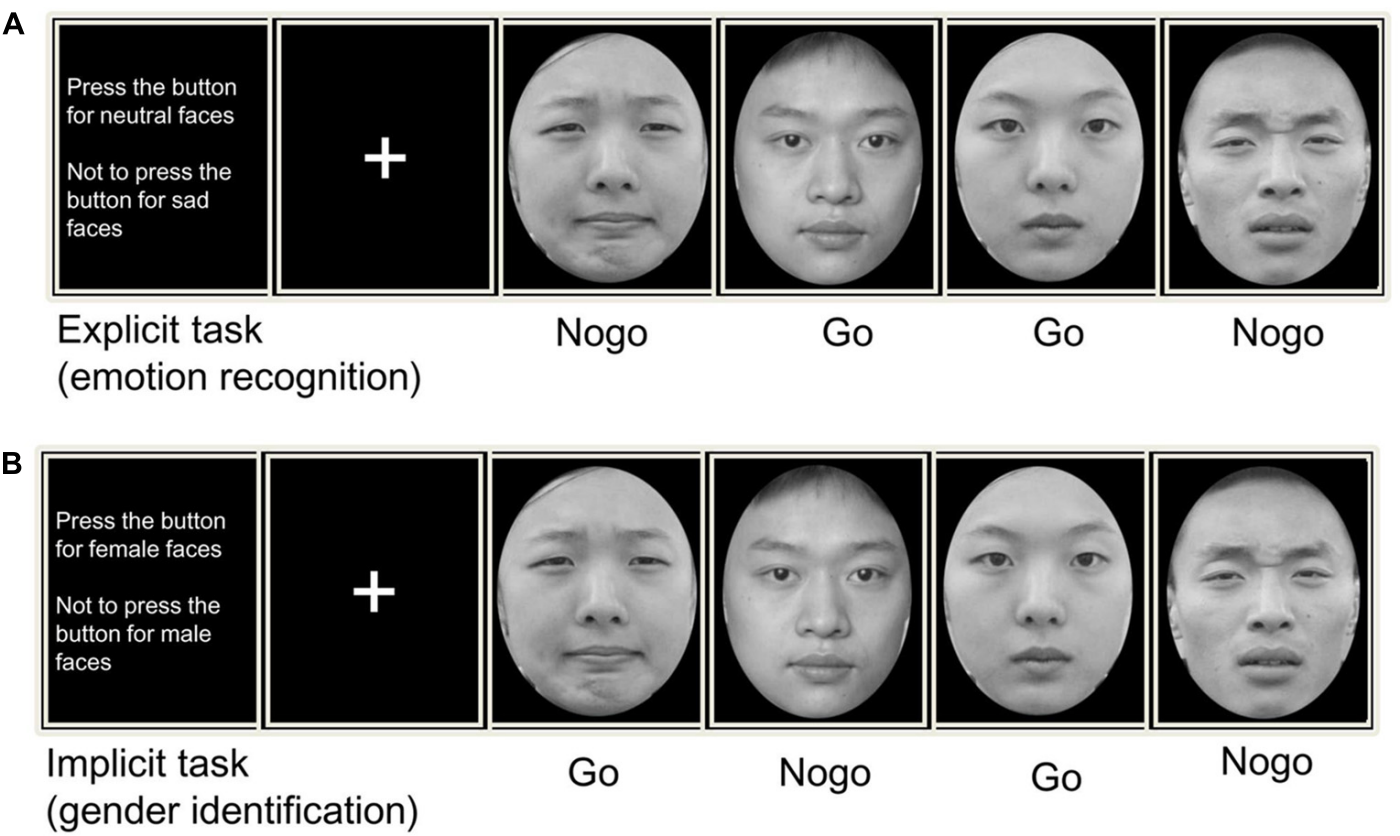
Trial design for **(A)** an explicit and **(B)** an implicit emotional Go/No-go tasks. In explicit task, subjects pressed a response button or inhibit their behavior according to the facial expression (sad/neutral). While in implicit task, subjects made their motor actions based on the facial gender (male/female).

Each trial was initiated by a small gray cross that was displayed for a variable duration (200–400 ms) on the black background. An emotional face appeared at the center of the screen for 1,000 ms. The participants were instructed to respond as quickly as possible on the promise of accuracy after the face was presented. Each response was followed by a blank screen presented for 1,200 to 1,500 ms, before the next trial began. The experimental procedure is presented in **Figure 1**. A training session including 20 trials was incorporated before the formal experiment. The stimuli included in the training session were different from those used in the main experiment. We compiled and executed all programs using E-Prime software (Psychology Software Tools, Inc., Pittsburgh, PA, USA). The independent variables of the present experiment were task (explicit and implicit), emotion (sad and neutral), trial type (go and no-go), and group (GAD and control). We used a multivariate analysis of variance for data analysis.

### Event-Related Potential Recording

Electroencephalography (EEG) was recorded from 64 scalp sites using tin electrodes mounted on an elastic cap (Neuro Scan, Sterling, VA, USA) according to the international 10/20 system. The system was grounded with a forehead electrode. All EEG signals were referenced to the left mastoid and were re-referenced off-line to the average of the left and right mastoids. Vertical electro-oculogram (EOG) data were recorded supraorbitally and infraorbitally in the left eye. Horizontal EOG data were recorded as the left versus right orbital rim. EEG and EOG activity was amplified with a 0.01–100 Hz band-pass filter and continuously sampled at 500 Hz/channel. All electrode impedances were maintained below 5 kΩ. Ocular artifacts were removed from the EEG signals using a regression procedure implemented in Neuroscan software (Semlitsch et al., 1986). Trials with remaining EOG artifacts, amplifier clipping artifacts, or peak-to-peak deflections exceeding ±100 μV were excluded from averaging. The EEG activities during correct responses in each condition were aligned and averaged separately. The ERP waveforms were time-locked to the onset of the face stimuli, and the average epoch was 1,200 ms, including a 200 ms pre-stimulus baseline.

### Behavior Result Analysis

We used signal detection theory to analyze the behavioral results. Signal detection theory is a method that discerns signal from noise. It assumes that the perceiver has a distribution of internal responses for both signal and noise (Snodgrass and Corwin, 1988; Stanislaw and Todorov, 1999). Based on this theory, we investigated participant’s discrimination accuracy (distance between signal and noise distributions) and decision bias (tendency to respond ‘signal’ or ‘noise’). The discrimination accuracy *d*′ was defined as *d*′ = (μ_s_–μ_n_)/σ, where μ_s_ is the mean of signal distribution and μ_n_ is the mean of noise distribution, and σ is the common standard deviation of both distributions. It is calculated as *d*′ = Z_hit rate_–Z_false alarm rate_, with four possible outcomes: hit (signal present and subject’s response is ‘yes’), miss (signal present and subject’s response is ‘no’), false alarm (signal absent and subject’s response is ‘yes’), and correct rejection (signal absent and subject’s response is ‘no’). Decision bias β was defined as β = f_s_(λ)/f_n_(λ), where f_s_(λ) is the height of the signal distribution at a given criterion λ and f_n_(λ) is the height of the noise distribution at the same λ. It was calculated as β = exp (*d*′ ×*C*), where *C* = –(Z_hit rate_ +Z_false alarm rate_)/2. Because the data set does not meet the Gaussian distribution and all comparisons were based on an *a priori* hypothesis for small samples, we used non-parametric tests. We used two independent sample tests to compare the d′ and β variables between the two groups.

### ERP Measure and Analysis

In this study, P1, VPP, N170, N2, and P3 components were measured and the peak and latency (P1, VPP, N170, N2, and P3: from stimulus onset to the peak of each component) and amplitudes (P1, VPP, and N170: baseline to peak; N2 and P3: mean amplitudes) were analyzed. According to the topographical distribution of grand-averaged ERP activity and previous studies (Williams et al., 2006; Righart and de Gelder, 2007; Yuan et al., 2008; Yu et al., 2009), different sets of electrodes sites were chosen for these components. We selected the following nine electrode sites for statistical analysis of the VPP (120–220 ms), N2 (220–320 ms), and P3 components (450–550 ms and 550–650 ms): F3, C3, P3, Fz, Cz, Pz, F4, C4, and P4. The P7, PO7, P8, and PO8 sites were used for statistical analysis of N170 (120–220 ms), and O1, OZ, and O2 were used for statistical analysis of P1 (60–140 ms). A 5-way mixed design ANOVA on the amplitude and latency of each component was conducted with task (two levels: implicit, explicit), emotion (two levels: neutral, sad), trial type (two levels: go and no-go) and electrode as within subject factors, and group (anxiety and control) as between subject factors. *P*-values were corrected by Greenhouse–Geisser correction. We used a Bonferroni method for multiple comparisons.

### Source-Localization Analysis

The sLORETA analysis was applied to calculate the cerebral generators of response inhibition (Pascual-Marqui, 2002). sLORETA is a three-dimensional discrete linear solution that has been frequently used for EEG source analysis. sLORETA is used to estimate current density distributions restricted to the cortical gray matter and the hippocampus in the digitized MNI atlas with 6,239 voxels at a spatial resolution of 5 mm. The sLORETA method has been shown to produce results that coincide with those provided by other brain imaging methods using equivalent paradigms (Dierks et al., 2000; Vitacco et al., 2002; Mulert et al., 2004; Pizzagalli et al., 2004).

To identify the different neural responses of response inhibition between the anxiety and control groups, we compared voxel-based whole brain sLORETA images between the groups during the no-go condition based on statistical non-parametric mapping (SnPM) methodology (Nichols and Holmes, 2002).

## Results

### Behavioral Performance

The response accuracy and response time were presented in **Table 2**. We used signal detection theory to analyze response accuracy. The ANOVA analysis revealed that GAD patients had significantly smaller *d*′ values compared to the control group when asked to respond to negative faces and inhibited responses to neutral faces under the explicit task (*t* = 2.82, *P* < 0.05), indicating diminished ability under this task. The differences in *d*′ between the two groups did not reach significance in other conditions. We did not observe a significant difference in β values between the two groups in any condition. It is worth noting that *d*′ and β are two independent measures; that is, discrimination accuracy does not correlate with decision bias. Our results demonstrated significant impairment in discrimination accuracy in response to negative faces and inhibited responses to neutral faces indexed by *d*′, yet there was no significant deficit in likelihood ratio decision bias measured by β under the explicit task. In addition, the statistical analysis of *d*′ and β showed that the difference between the implicit task and explicit task were not significant in both groups.

**Table 2:** Amplitudes in P, N170, VPP, N2, P3 segment of the study participants.

	Explicit task				Implicit task			
	Negative		Neutral		Negative		Neutral	
	Go	No-go	Go	No-go	Go	No-go	Go	No-go
P1 (GAD)	5.0 (0.7)	5.6 (0.7)	5.3 (0.7)	5.4 (0.7)	5.0 (0.7)	5.4 (0.7)	5.0 (0.7)	5.4 (0.7)
P1 (CON)	5.5 (0.7)	5.8 (0.7)	5.5 (0.7)	5.8 (0.7)	5.1 (0.7)	5.5 (0.8)	5.1 (0.7)	5.8 (0.7)
N170 (GAD)	–2.8 (0.6)	–2.7 (0.6)	–2.8 (0.6)	–2.6 (0.6)	–3.8 (0.6)	–3.8 (0.6)	–3.9 (0.5)	–3.8 (0.6)
N170 (CON)	–1.1 (0.6)	–1.5 (0.6)	–1.8 (0.6)	–1.4 (0.6)	–2.1 (0.6)	–2.4 (0.6)	–2.1 (0.5)	–2.0 (0.6)
VPP (GAD)	9.0 (0.8)	9.4 (0.9)	8.3 (0.8)	9.0 (0.8)	8.3 (0.8)	8.4 (0.9)	7.9 (0.8)	8.0 (0.8)
VPP (CON)	6.9 (0.8)	7.2 (0.8)	6.0 (0.8)	6.1 (0.8)	6.0 (0.8)	6.1 (0.9)	5.8 (0.8)	5.6 (0.9)
N2 (GAD)	4.4 (0.7)	4.2 (0.8)	3.8 (0.8)	4.3 (0.7)	2.7 (0.7)	2.3 (0.8)	2.6 (0.7)	2.1 (0.8)
N2 (CON)	3.9 (0.7)	3.4 (0.8)	2.7 (0.8)	2.6 (0.7)	1.8 (0.7)	1.7 (0.8)	1.8 (0.7)	1.5 (0.8)
P3 (GAD)	5.8 (0.7)	7.9 (0.9)	6.1 (0.7)	6.5 (0.8)	4.6 (0.7)	5.8 (0.9)	4.8 (0.8)	6.0 (0.9)
P3 (CON)	7.0 (0.7)	8.7 (0.9)	5.5 (0.7)	6.8 (0.8)	4.0 (0.7)	6.4 (0.9)	4.0 (0.8)	6.6 (0.9)

*Amplitudes in microvolt. All values expressed in mean (SD). GAD, general anxiety disorder; CON, control*.

The repeated-measures ANOVA showed that the reaction times undergo conditions were significantly affected by task and emotion (*F*_1,36_ = 10.28, *P* < 0.005; *F*_1,36_ = 13.59, *P* < 0.005). The reaction times were significantly shorter for the explicit task than for the implicit task and were shorter under negative conditions than under neutral conditions. Additionally, the three-way interaction was also significant (*F*_1,36_ = 5.95, *P* < 0.05). We further explored the 3-way interaction effect by splitting the task factor into two separate 2-way ANOVA analyses. The 2-way ANOVA analyses with emotion as a within-subject factor and group as a between-subject factor showed that the 2-way interaction effect in the implicit task was not significant (*F*_1,36_ = 0.14, *P* > 0.5), while notably it was significant in the explicit task (*F*_1,36_ = 5.25, *P* < 0.05). We, in turn, explored the group effect in negative and neutral conditions, respectively, with two separate *t*-tests. The results revealed that GAD patients showed a significantly shorter reaction time than the control group under the negative condition during the explicit task (*t*_36_ = 2.21, *P* < 0.05). The difference between the two groups under neutral (*t*_36_ = 0.49, *P* > 0.5) conditions was not significant.

### ERP Data Analysis

#### P1

**FIGURE 2:**
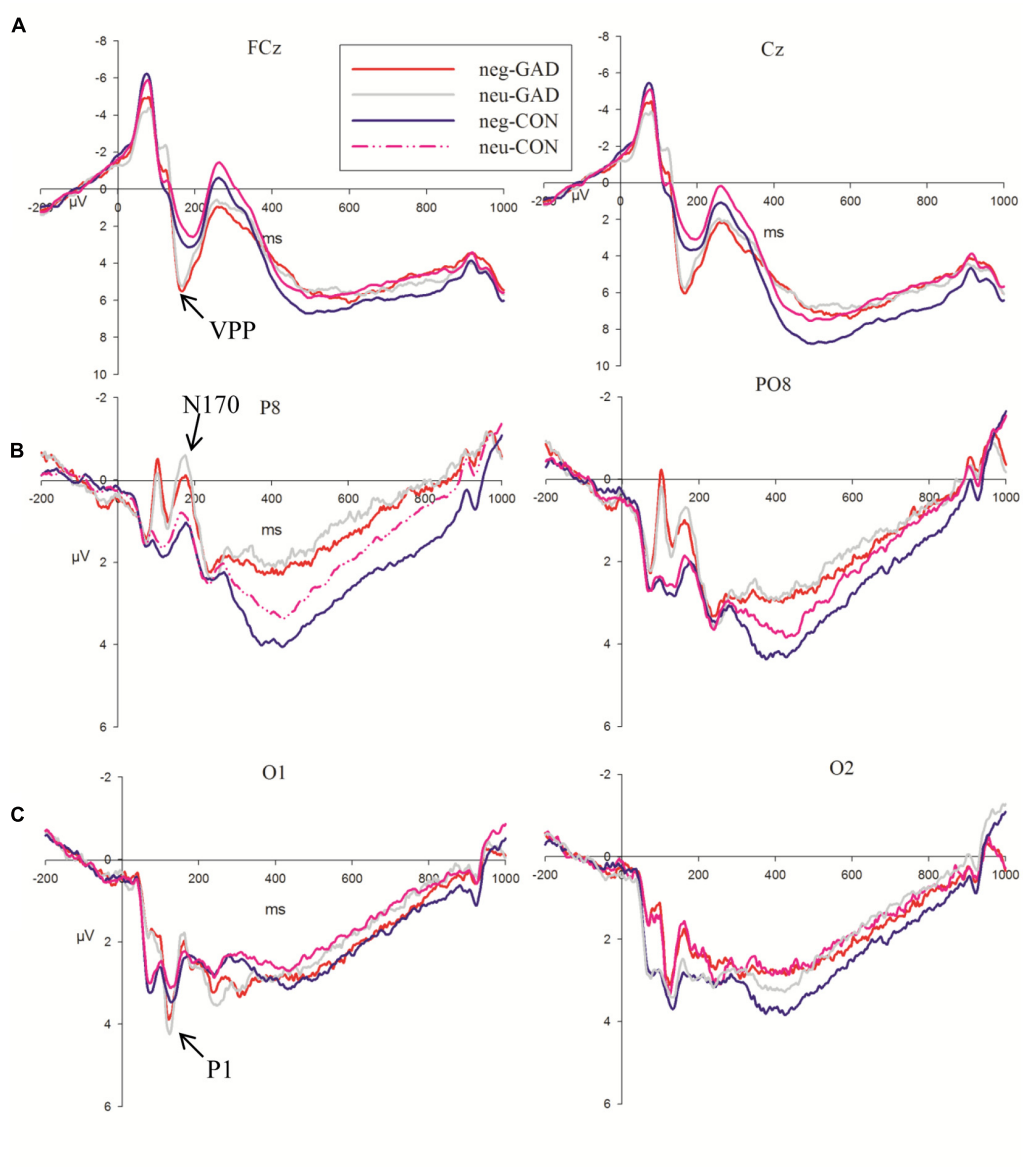
Grand averages evoked by face stimuli of the two groups at FCz, Cz, P8, PO8, O1, and O2 sites. The shaded area indicates the time windows in which the P1 **(A)**, VPP **(B)**, N170 **(C)** were analyzed (GAD, general anxiety disorder; CON, control).

The 5-way ANOVA analyses with task, emotion, trial type, and electrode as within-subject factors and group as the between-subject factor did not reveal any significant main effect or interaction effect in P1 amplitudes. P1 latency showed a significant main effect at task (*F*_1,36_ = 5.15, *P* < 0.05) and group (*F*_1,36_ = 6.16, *P* < 0.05). A shorter P1 latency was elicited in the implicit task (115.89 ms) compared to the explicit task (119.58 ms). The anxiety group (112.68 ms) showed a shorter P1 latency than the control group (122.79 ms). As P1 latency showed task and group main effects, we present the P1 result average across task, emotion and group in **Figure 2A**.

#### N170

N170 amplitudes showed a significant main effect for task (*F*_1,36_ = 24.36, *P* < 0.001). The faces in the explicit task (–2.98 μV) elicited larger N170 amplitudes than that in the implicit task (–2.12 μV). The group × electrodes interaction effect showed that the anxiety group (–4.14 μV; –4.09 μV) showed larger amplitudes than the control group (–1.92 μV; –1.16 μV) at P8 and PO8 electrode sites, respectively (*F*_1,36_ = 6.24, *P* < 0.05; **Figure 2B**). The amplitude differences between the two groups at P7 and PO7 electrode sites were not significant (*F*_1,36_ = 0.14, *P* > 0.7). The N170 latency showed a significant group (*F*_1,36_ = 4.96, *P* < 0.05) main effect. The anxiety group (159.48 ms) showed a shorter N170 latency than the control group (168.51 ms).

#### VPP

Task, emotion, and electrodes had an effect on the VPP amplitude (*F*_1,36_ = 14.11, *P* = 0.001; *F*_1,36_ = 30.13, *P* < 0.001; *P* < 0.001; *F*_1,36_ = 3.21, *P* < 0.05; The VPP amplitude was larger in the explicit task (7.78 μV) than in the implicit task (7.03 μV). Sad faces (7.69 μV) elicited a larger amplitude than neutral faces (7.12 μV). The amplitudes of FCz (8.06 μV, *P* < 0.05) and Cz (8.29 μV, *P* < 0.05) were significantly larger than the other sites. Moreover, we detected that the group main effect was marginally significant (*F*_1,36_ = 3.85, *P* = 0.057). The VPP amplitude in the anxiety group (8.58 μV) was somewhat larger than that in the control group (6.23 μV; **Figure 2C**).

The main effects of emotion and electrodes in VPP latency were significant (*F*_1,36_ = 11.90, *P* = 0.001; *F*_1,36_ = 3.89, *P* < 0.05). The VPP latency elicited by sad faces (182.96 ms) was shorter than that elicited by neutral faces (186.77 ms). The latency of FCz (180.33 ms, *P* < 0.05) and Cz (180.15 ms, *P* < 0.05) was significantly shorter than the other sites.

#### N2

**FIGURE 3:**
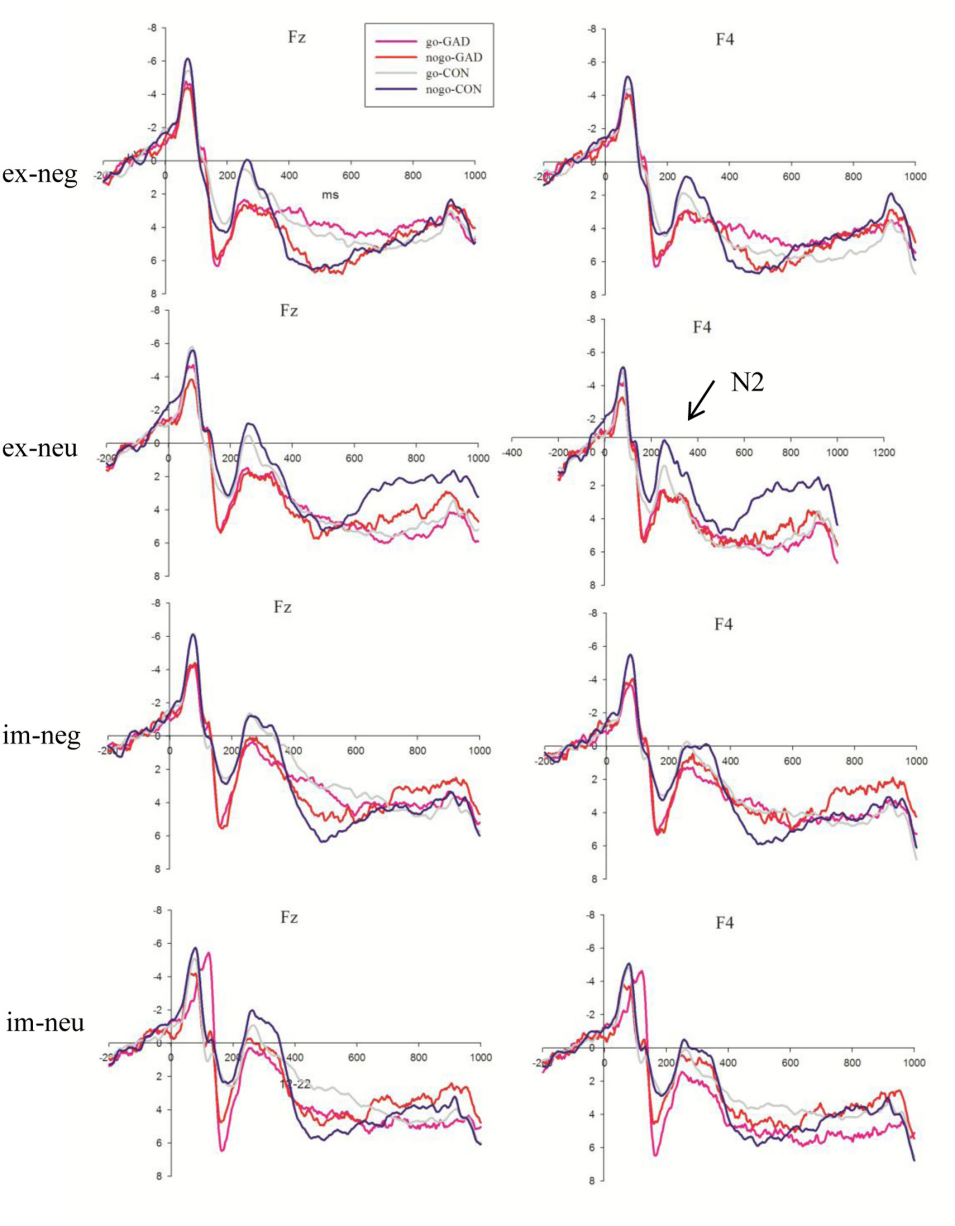
The N2 average waves evoked by go and no-go stimuli in GAD group (red lines; pink lines) and control group (blue lines; gray lines) in explicit negative, explicit neutral, implicit negative, implicit neutral conditions at Fz, F4 sites (GAD, general anxiety disorder; CON, control; im, implicit; ex, explicit; neg, negative; neu, neutral).

The N2 amplitudes showed a significant main effect for task, emotion, and electrodes (*F*_1,36_ = 42.91, *P* < 0.001; *F*_1,36_ = 12.35, *P* = 0.001; *F*_8,288_ = 23.2 respectively, *P* < 0.001). The explicit task (3.71 μV) elicited larger amplitudes than the implicit task (2.11 μV). The amplitude elicited by sad faces (3.09 μV) was larger than that elicited by neutral faces (2.73 μV). The N2 amplitudes were larger at FC3 (1.54 μV, *P* < 0.05), FCz (0.56 μV, *P* < 0.001), and FC4 (1.82 μV, *P* < 0.05) than the other sites. We detected that the trial type and electrode interaction effect was significant (*F*_1,36_ = 3.97, *P* < 0.05). The simple analysis showed that the amplitudes elicited by no-go trials (1.02 μV) were larger than go trials (1.36 μV) at FCz and FC4 (*F*_8,288_ = 4.66, *P* < 0.05). The trial type effect was not significant at the other sites. More importantly, the task, trial type, electrode, and group interaction effect were significant (*F*_8,288_ = 2.66, *P* < 0.05; **Figure 3**). To clearly illustrate this interaction effect, we calculated the no-go/go difference waves to observe the dissimilarity between the anxiety and control groups.

The main effects of trial type in N2 latency were significant (*F*_1,36_ = 4.62, *P* < 0.05). The N2 latency elicited by go stimuli (300.74 ms) was shorter than that elicited by no-go stimuli (309.07 ms).

#### No-go/go Differences in Event-Related Potentials at the N2 Interval

**FIGURE 4:**
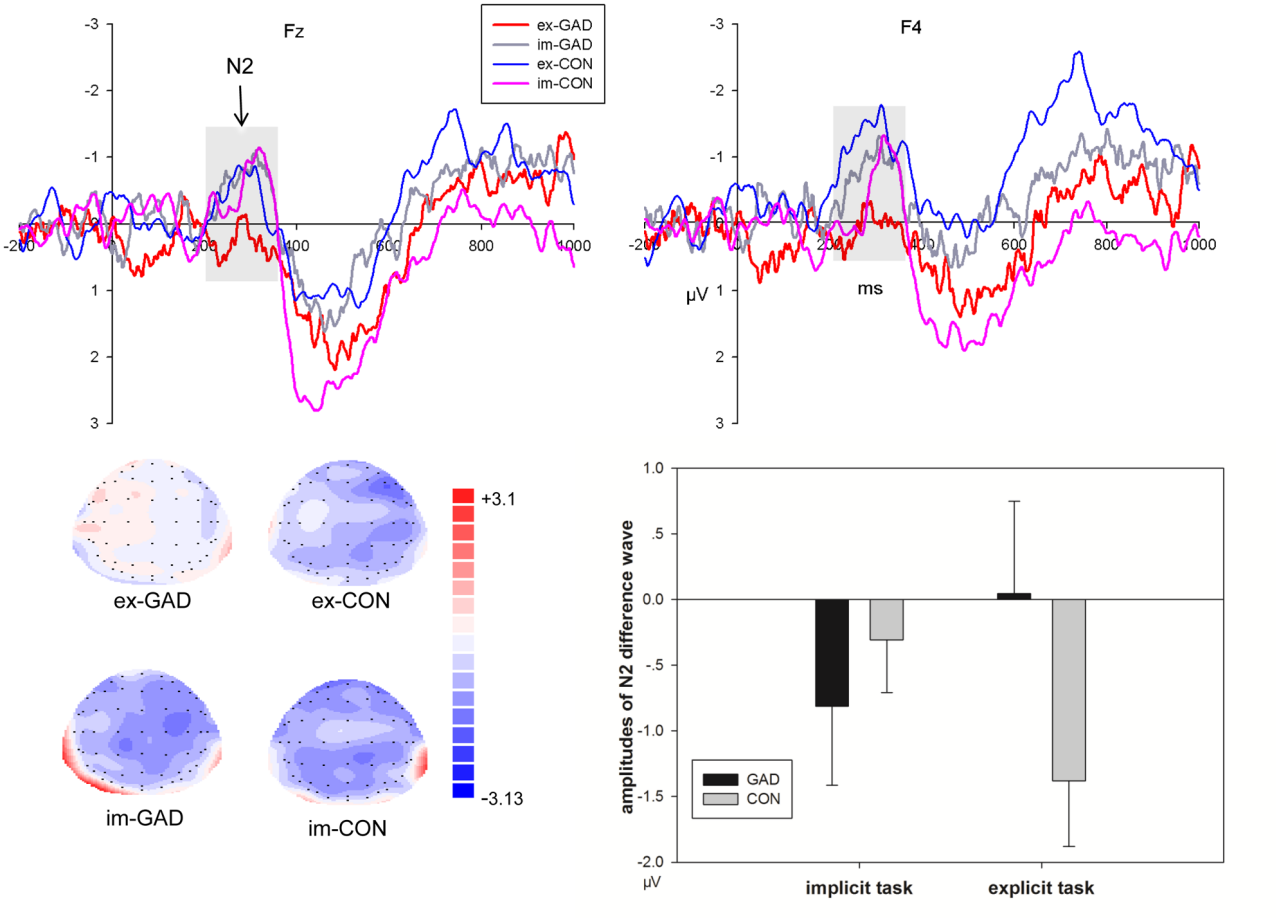
The N2 difference waves of No-go minus Go trials at Fz, F4 sites **(upper)** and corresponding amplitudes histogram **(bottom right)** and scalp topographies **(bottom left)** of the two groups during implicit and explicit tasks (GAD, general anxiety disorder; CON, control; im, implicit; ex, explicit).

*No-go/go differences in event-related potentials* were presented in **Figure 4**. The interaction effect between task, trial type, electrode, and group was significant on the amplitudes at the N2 interval. To expound the features of this interaction, we focused analysis on the difference wave of no-go minus go conditions. The 4-way multivariate ANOVA on the N2 amplitudes revealed significant task, electrode, and group interaction effects (*F*_8,288_ = 2.66, *P* < 0.05). The simple analysis revealed that the control group (–0.71 μV) showed larger amplitudes than the anxiety group (0.08 μV) in explicit tasks at the right fronto-central sites (*F*_1,36_ = 3.47, *P* = 0.07). The amplitudes showed a larger tendency in the GAD group (–0.7 μV) than in the control group (–0.2 μV), but the difference was not significant in the implicit task (*F*_1,36_ = 1.81, *P* > 0.1).

#### P3

**FIGURE 5:**
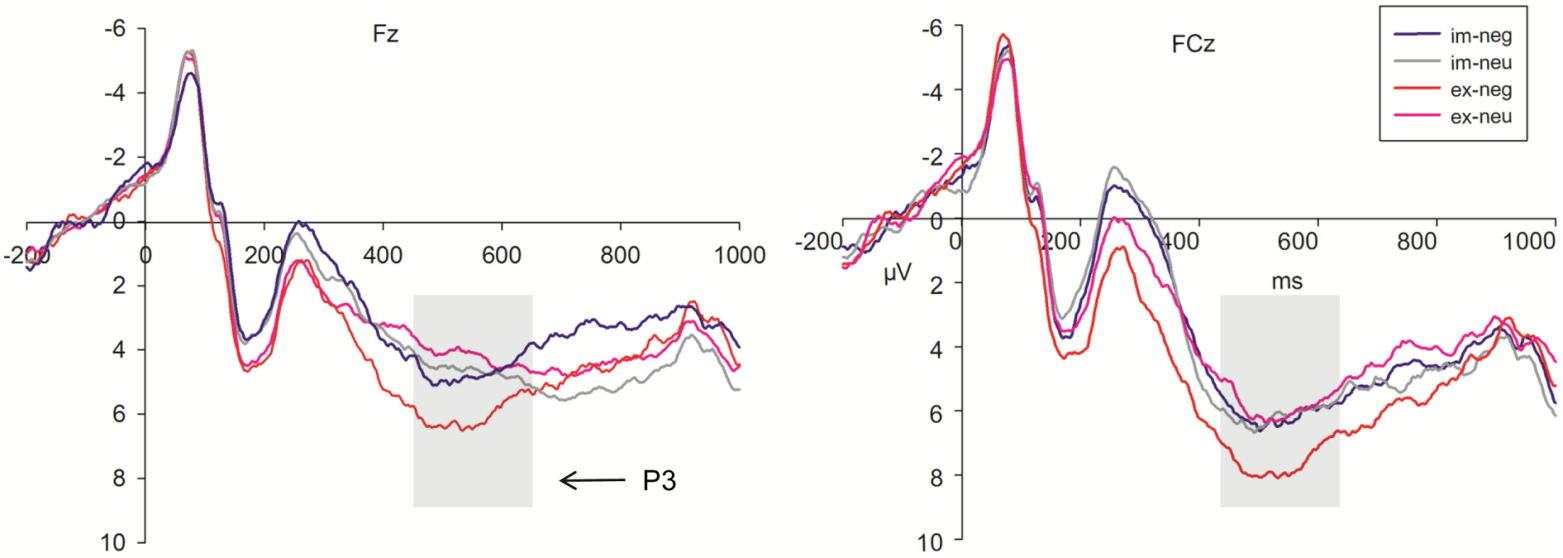
Grand averages evoked by negative and neutral stimuli under implicit task (blue lines; gray lines) and explicit task (red lines; pink lines) in No-go trials at Fz, FCz sites (im, implicit; ex, explicit; neg, negative; neu, neutral).

Because the time window was long, P3 was divided into two segmentations, 450–550 ms and 550–650 ms. During the 450–550 ms time window (**Figure 5**), P3 amplitudes showed a significant main effect for task, emotion, trial type, and electrode (*F*_1,36_ = 28.06, *P* < 0.001; *F*_1,36_ = 13.29, *P* = 0.001; *F*_1,36_ = 24.62, *P* < 0.001; *F*_8,288_ = 12.61, respectively, *P* < 0.001). The amplitudes were larger during the explicit task (6.80 μV) than in the implicit task (5.33). Sad faces (6.32 μV) elicited larger amplitudes than neutral faces (5.81 μV). No-go trials (6.87 μV) elicited larger amplitudes than go trials (5.26 μV). Pz (8.19 μV) elicited the largest amplitudes of P3. Cz (6.84 μV), C4 (6.74 μV), and P4 (6.29 μV) electrodes elicited larger amplitudes than the other sites. We found that the interaction effect between task, emotion, and trial type was marginally significant (*F*_1,36_ = 3.74, *P* = 0.06; **Figure 5**). The simple analysis revealed that no-go/P3 amplitudes elicited by sad faces (8.31 μV) were larger than those elicited by neutral faces (6.67 μV) in the explicit task. The no-go amplitude differences between sad (6.18 μV) and neutral (6.35 μV) conditions were not significant in the implicit task.

During the 550–650 ms time window, P3 amplitudes showed significant task, emotion, trial type, and electrode main effects (*F*_1,36_ = 29.55, *P* < 0.001; *F*_1,36_ = 7.32, *P* = 0.01; *F*_1,36_ = 41.64, *P* < 0.001; *F*_8,288_ = 13.93, respectively, *P* < 0.001). P3 amplitudes were larger in the explicit task (6.98 μV) than in the implicit task (5.61 μV). Sad faces (6.51 μV) elicited larger amplitudes than neutral faces (6.07 μV). No-go trials (7.21 μV) elicited larger amplitudes than in go trials (5.38 μV). The amplitudes at Cz (7.62 μV) and Pz (7.74 μV) were larger than the other sites.

The main effects of trial type in P3 latency were significant (*F*_1,36_ = 31.95, *P* < 0.001). The P3 latency was longest at Cz (611.98 ms).

#### Relationship between Clinical Characteristics and Task-Related Measures

Significant correlations were not found between the clinical measures (HAMA) and the discrimination accuracy, which also occurred in the relationship of clinical measures and no-go/N2 amplitudes in the explicit task. We did not find correlations between no-go/N2 and behavior accuracy.

#### Source-Localization Data

**FIGURE 6:**
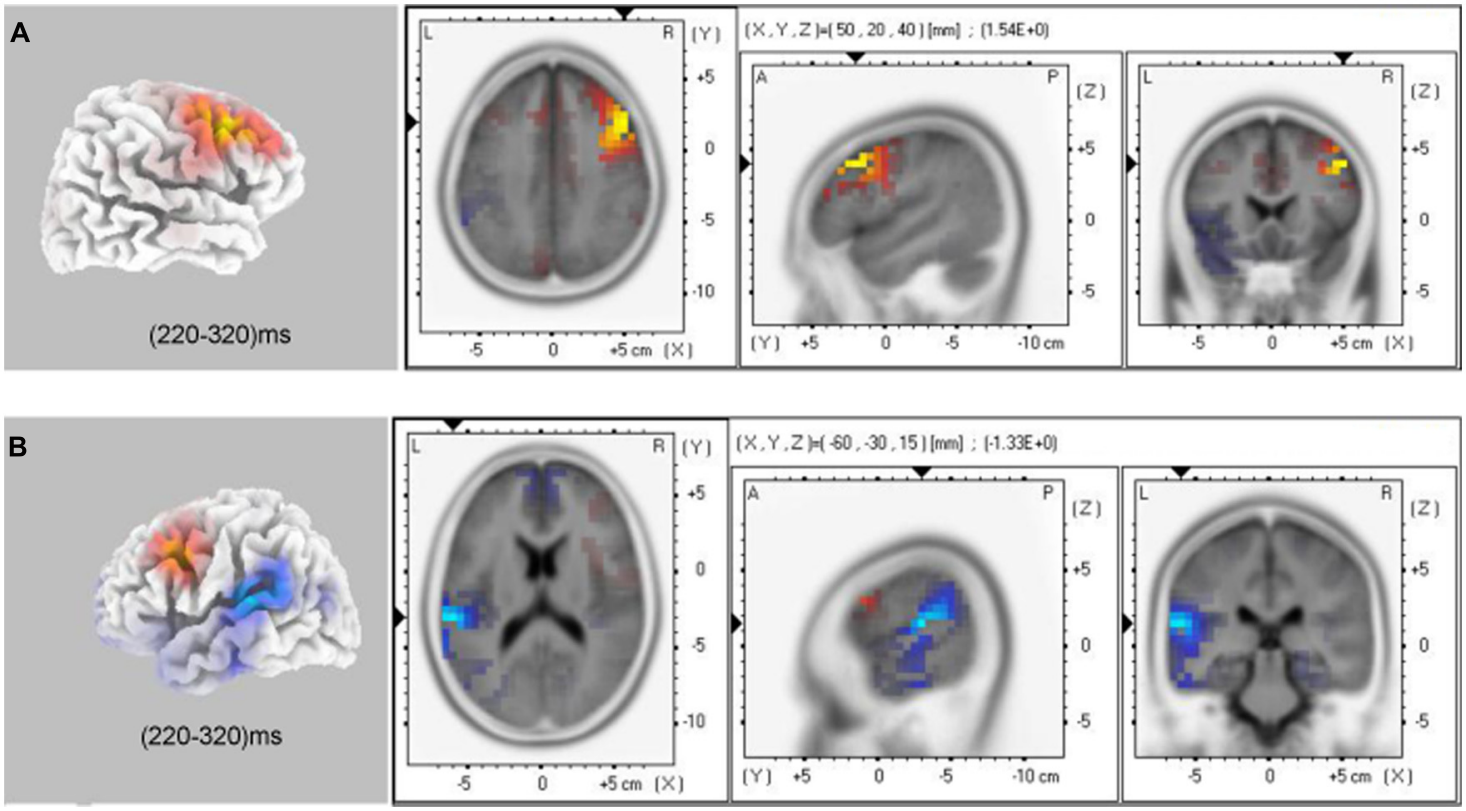
sLORETA solutions to non-parametric randomization tests on N2 components showing voxels in which the CON group > GAD group contrast **(A)** and GAD group > CON group contrast **(B)** were significant (*P* < 0.05; GAD, general anxiety disorder; CON, control).

The voxel-based whole-brain sLORETA images for control and anxiety groups under no-go conditions at the N2 interval were compared using non-parametric randomization tests in order to identify the cortical regions involved. As hypothesized, the brain regions involved in response inhibition processing varied between the control and anxiety groups in the explicit (*t*_36_ = 1.29, *P* < 0.05) and implicit tasks (*t*_36_ = 1.29, *P* < 0.05). The current source density (CSD) in the right DLPFC (Brodmann 9, max values obtained at *x* = 50, *y* = 20, and *z* = 40) in the control group was larger than in the anxiety group. Moreover, the current source density in the left superior temporal gyrus (lSTG, Brodmann 42, max values obtained at *x* = –60, *y* = –30, and *z* = 15) and left inferior parietal Lobule (lIPL, Brodmann 40, max values obtained at *x* = –60, *y* = –45, and *z* = 20) were larger in the anxiety group than in the control group in the implicit task (*t*_36_ = –1.21, *P* < 0.05; **Figure 6**).

## Discussion

The present study aimed to examine the neural substrates of response inhibition to sad faces across implicit and explicit tasks in clinical GAD individuals using high time resolution ERP methods. The GAD group showed smaller amplitudes of no-go/go difference waves across sad and neutral emotions specifically in the explicit task rather than in the implicit task at the N2 interval when compared to controls. The source localization of no-go/N2 components showed a lower CSD of rDLPFC in both implicit and explicit tasks and a higher CSD of lSTG and lIPL in implicit tasks in GAD individuals compared to the control group. Thus, our results provide further evidence for the dissociation at a neural level of response inhibition to sad faces between implicit and explicit tasks.

As shown in **Figure 2**, at the 60–140 ms interval, a posterior P1 component reflecting early visual processing was induced by facial stimuli. As expected, the present study observed that the GAD group showed a shorter latency than the control group, irrespective of task, and emotional conditions. In addition, the faces in the present study induced prominent face-specific N170 and VPP components. The GAD group showed larger N170 and VPP amplitudes relative to the control group. This result was consistent with previous studies, which reported that N170 and VPP can be modulated by attention (Holmes et al., 2003; Mohamed et al., 2009). As usual, GAD patients were sensitive to facial stimuli and they allocated more attention resources to face processing relative to the control group (Douilliez et al., 2012; Peschard et al., 2013). The result was similar to previous studies reporting that anxious individuals exhibit a faster attention capture to facial expression and evince faster latency in early ERP components (Russell, 1989; Peschard et al., 2013). The above results suggest that the GAD group automatically shows an attention bias to face processing at an early stage.

More remarkable, this study observed a significant interaction effect between the task and group for the no-go/go difference wave at the 220–320 ms interval. As indicated in **Figure 4**, the subtraction of ERPs elicited by go stimuli from those elicited by no-go stimuli generated a clear no-go related N2 component during this stage. The amplitudes of no-go related N2 were significantly smaller in the GAD group compared to the control group in the explicit task, irrespective of the emotion type. Previous ERP studies also found that anxious children showed decreased no-go/go difference wave amplitudes during the N2 stage (Hum et al., 2013) and no-go/N2 was negatively related to anxiety-related personality traits (Sehlmeyer et al., 2010). The present study further found that GAD patients showed decreased no-go/go difference waves at the N2 interval in the explicit task compared to the control group. As previously mentioned, the no-go related N2 amplitude was accepted as the index of conflict monitoring and for the increased attention engagement that forms the basis for the subsequent process of inhibition (Yuan et al., 2008). In explicit task, facial expression is within the voluntary attention scope and is directly processed. In implicit task facial expression is within the involuntary attention scope, and therefore is incidentally processed. Thus, the attention resource for emotion processing is distinct between the two conditions. The difference of N2 amplitudes between GAD and control was dissociated between the two tasks. These results suggested that response inhibition of GAD individuals were modulated by attention at the conflict monitoring stage. From these results, we concluded that the processing of response inhibition was interrupted in GAD when subjects were asked to respond to facial expressions. In this situation, GAD patients were more sensitive to the directly processed facial expressions and devoted more attention resources to facial expression processing. According to the shared resources theory, emotional processing requires cognitive resources and consumes the common pool of such resources with top–down cognitive control (Pessoa, 2009). Thus, the current direct goal of response inhibition was interfered by increased response-competition of the task-irrelevant distracters (Berggren and Derakshan, 2013). These results were consistent with previous studies that indicated that anxiety shows a response inhibition deficit using emotional distracters (Cisler and Koster, 2010; Berggren and Derakshan, 2013). In addition, GAD also showed decreased amplitudes of N2 difference wave in neutral condition in explicit task. Previous study have suggested that social anxiety disorder patients tend to interpret neutral and other ambiguous emotional stimuli negatively (Winton et al., 1995). Neuroimaging studies found that amygdala showed a different activation pattern in response to neutral faces compared to controls (Cooney et al., 2006; Veit et al., 2006). In the present study, GAD patients may also interpret neutral faces negatively and showed abnormal response inhibition to neutral faces.

In contrast to the explicit task, the implicit task showed somewhat larger N2 amplitudes in the GAD group compared to the control group even though the difference was not significant. In the implicit task, participants were introduced to responses or suppressed their responses according to face gender. The attention was diverted from facial expressions. Gross has proposed that the emotional effect on cognition was weakened when attention was disengaged from the emotional aspects of stimuli (Gross, 2001; Ochsner and Gross, 2005). Consistent with Gross’ work, the present results indicated that response inhibition to social negative stimuli in GAD individuals could be regulated by attention resources. When introduced to disengage attention from negative events to non-emotional aspects, GAD individuals showed an improved ability to inhibit responses to social information. These results support attention bias modification treatment therapy in GAD individuals. The attention bias modification means training attention to tend to positive or neutral stimulus and to avoid negative stimulus. A large number of studies reported that after attention bias modification treatment, the anxiety symptom in GAD decreased significantly (Browning et al., 2010; Hakamata et al., 2010; Hallion and Ruscio, 2011; MacLeod and Mathews, 2012). The present study further provides neural evidence that the cognitive processing was modified in GAD when asked to divert attention from emotional to non-emotional aspects. However, the present results were inconsistent with previous studies that reported that facial processing was not affected by task instructions in social anxiety individuals. The participants showed attention bias to faces in both implicit and explicit tasks (Peschard et al., 2013). This may be because the previous study only investigated the modulated effect of task instruction on face processing but not the effect of the emotion on response inhibition, as in the present study. Another reason was that the participants in that study had subclinical social anxiety and the present study included general anxiety individuals. There could be differences in neural mechanisms of interaction of emotion and cognition between subclinical social anxiety and GAD.

By using signal detection theory, we found that GAD showed poor discretionary accuracy of *d*′ compared to controls when asked to respond to sad faces and inhibit motor responses to neutral faces in the explicit task. This was interpreted as emotional faces inducing an amplified vigilance in GAD. Thus, when asked to response to the sad faces, they made a faster response to the stimuli and the error rate increased. The results were consistent with previous studies, which also found anxiety individuals showed faster responses to threatening and happy faces (Bradley et al., 1999; Rossignol et al., 2005). Several studies reported that anxiety individuals showed attention bias to social threatening stimuli (fearful or angry faces; Bradley et al., 1999; Fox, 2002; Pergamin-Hight et al., 2014). The present study further found that GAD patients also showed attention bias to sad faces when processed explicitly. In addition, we calculated the correlation between discrimination accuracy and clinical characteristics and response times and clinical characteristics. However, the correlation was not significant. The results may indicate that attention bias to sad faces in the explicit task is an endophenotype of GAD and unrelated to the clinical characteristics. While in the implicit task, there were no significant differences in behavioral performances between GAD patients and the control group. Previous studies have revealed that, although anxious individuals showed reduced response efficiency (i.e., the pattern in which cognitive resources are utilized to achieve the desirable performance outcome), the performance effectiveness (i.e., the ability to perform the task) was well preserved relative to controls (Righi et al., 2009; Ansari and Derakshan, 2011; Berggren and Derakshan, 2013). The reduced cognitive efficiency may be ameliorated by strategies such as a compensatory effort (Eysenck et al., 2007). However, other studies reported that anxious individuals showed impaired performance effectiveness because of interruption by task-irrelevant sad faces (Bishop, 2009; Ansari and Derakshan, 2011; Basten et al., 2011). The present study found that in the implicit task there was no difference in discrimination accuracy and response time. Performance effectiveness in the explicit task was decreased in the GAD group. According to the shared resources theory, the processing of facial expressions occupied even more cognitive resources and the remaining resources for compensatory efforts on the current direct goal were limited (Pessoa, 2009). Thus, the present results reconcile the conflict by showing that that the performance effectiveness was well preserved in implicit task, but not in explicit task.

After a conflict monitoring process indicated by N2, an inappropriate response was inhibited at the P3 stage (Bokura et al., 2001; Albert et al., 2010). Similar to the results of our previous study (Yu et al., 2014), we observed a task, emotion, and trial type interaction effect at the P3 interval (see details in **Figure 5**). In the explicit task, inhibiting negative faces elicited larger P3 amplitudes than neutral faces. However, the difference in no-go/P3 amplitudes between sad and neutral conditions was not significant during the implicit task. In this task, participants were instructed to respond to or inhibit their response according to facial gender, so facial expressions were task-irrelevant stimuli and their attentions were diverted from facial expressions. The intensity of negative emotion was decreased when the attention was disengaged from the emotional stimuli in the implicit task compared to the explicit task (Pessoa et al., 2005; Schonfelder et al., 2013), thus resulting in a reduced emotional effect on response inhibition.

Using sLORETA source localization analysis during the no-go/N2 time window, we observed that the GAD group displayed decreased CSD of rDLPFC compared to the control group across implicit and explicit tasks (**Figure 6**). Neuroimaging research in humans identified that the rDLPFC, as part of the rIFC, plays a critical role in response inhibition, task switching, updating, and other cognitive control functions (Verbruggen et al., 2010; Meyer et al., 2011). Convergent reports have emphasized the key role of DLPFC in the pathophysiology of GAD individuals (Meyer et al., 2011). Consistently, the present results further revealed a decreased ability of inhibition to task-irrelevant items and the dysfunction of rDLPFC may be an important cognitive and biological mechanism of GAD individuals. In addition, we observed that, in the implicit task, GAD individuals showed a significantly higher CSD in the junction of lSTG and lIPL (BA 42/40) relative to the control group. Previous neuroimaging researches reported that STG and IPL were part of a network for voluntary attention control and were involved in the processing of attention selection, attention shifting, and working memory in the general population (Hopfinger et al., 2000; Shapiro et al., 2002; Achiron et al., 2013). The abnormal structure and function in these areas led to impaired cognitive performance (Behrmann et al., 2004; Achiron et al., 2013). In the present study, the lSTG and lPLbrain regions were involved in allocating more attention resources to perform the implicit tasks and compensate for the function of rDLPFC in GAD individuals. However, the source localization method was based on the proposition from a mathematical, and not a physiological, standpoint. Future research employing a wide range of experimental tasks and designs, as well as brain imaging methodologies that may improve the spatial resolution, such as fMRI, is needed to substantiate and extend these findings.

The present study has several limitations. First, we only used the sad expressions as experimental stimuli and how other basic emotions interact in response inhibition in GAD across implicit and explicit tasks warrants investigation. Second, the dissociation of electrophysiological and behavioral difference between implicit and explicit tasks may due to the difficulty difference. To investigate the difficulty difference between the two tasks, we used signal detection theory to analyze the behavior performance. The discrimination accuracy and decision bias did not show differences between the implicit and explicit tasks in both groups. However, the statistical methods could not analyze this objective error well. We will manipulate the difficulty of the two tasks as a variable in later experiments.

## Conclusion

The present results demonstrated that GAD individuals show deficits when inhibiting responses to sad faces in an explicit task rather than in an implicit task. The amplitudes of no-go/go difference waves at the N2 interval were significantly smaller in GAD individuals than in controls during the explicit task, but showed a reversed trend during the implicit task. In addition, the results confirmed that GAD participants displayed a smaller rDLPFC in the no-go condition, which indicated that dysfunction of rDLPFC may play a critical role in the biological mechanism of GAD. The larger lSTG/lPL activation in GAD individuals in the implicit task may compensate for the dysfunction of rDLPFC. These results provided evidence for attention bias modification treatment in GAD individuals.
